# Dr. Roesch on the Nature and Treatment of Scrofula

**Published:** 1842-08-27

**Authors:** 


Cod liver oil retains its popularity in some parts of the continent, as a remedy for scrofula, disgusting as it is.

*On the Nature and Treatment of Scrofula.* By DR. ROESCH.—After enumerating the different forms of scrofulous cachexia, Dr. Roesch arrives at the conclusion that scrofulous affections are produced by an excess of acid matters in the fluid of the body. Agreeing with the ancient physicians in his theory of the disease, he recommends their plan of treatment, viz., absorbents, alkalies, and fat or oily matters. He says he has observed that, in those countries where the children get a quantity of lard and other fat matters with their food, that scrofula is extremely rare. Cod-liver oil is, therefore, according to him, one of the most suitable remedies to administer in this disease, seeing it possesses the rare properties of being at once a stimulant, a roborant, an antacid, and nutrient. He considers that the iodine in it will have a very secondary effect, the other properties of the oil being the most valuable.—*Ed. Med. and Surg. Journ., from Haeser's Archiv fur die Gesammte Medicin.*

